# Functional interactions between glutamate receptors and TRPV1 in trigeminal sensory neurons

**DOI:** 10.1186/1744-8069-10-S1-O13

**Published:** 2014-12-15

**Authors:** Jin Y Ro, Man-Kyo Chung, Jong S Lee, Jami L Saloman, John Joseph

**Affiliations:** 1 Department of Neural and Pain Sciences, University of Maryland School of Dentistry, Baltimore, MD 21201, USA

## Background

Elevated glutamate levels within injured muscle exacerbate pain conditions and modulate functional properties of muscle nociceptors via peripheral glutamate receptors. Direct injections of capsaicin in muscle tissue significantly lower mechanical thresholds, and the blockade of TRPV1 attenuates mechanical hyperalgesia resulting from eccentric muscle contraction. This study explores how the two receptor-channel systems that have been independently implicated in muscle pain and hyperalgesia interact together, which should offer novel perspectives on how various receptors and channels in nociceptors operate as ‘functional units’. We hypothesized that activation of peripheral glutamate receptors leads to TRPV1-dependent mechanical hyperalgesia via distinct intracellular signaling pathways.

## Results

In the masseter muscle, direct application of NMDA induced a time dependent increase in mechanical sensitivity, which was significantly blocked when the muscle was pretreated with a specific TRPV1 antagonist, AMG9810. Calcium imaging analyses further corroborated that NMDA receptors and TRPV1 in trigeminal ganglia (TG) functionally interact. In dissociated TG culture, application of NMDA resulted in phosphorylation of serine, but not threonine or tyrosine, residues of TRPV1 in a time course similar to that of the development of NMDA-induced mechanical hyperalgesia. The NMDA-induced phosphorylation was significantly attenuated by CaMKII and PKC inhibitors, but not by a PKA inhibitor. Consistent with the biochemical data, the NMDA-induced mechanical hyperalgesia was also effectively blocked when the muscle was pretreated with a CaMKII or PKC inhibitor. We further demonstrated that the activation of NMDA receptors specifically increased the phosphorylation of S800 (p-S800) of TRPV1 at cell surface membrane and that A-Kinase anchoring protein 150 (AKAP150) was required for NMDA-and PKC-mediated p-S800 of TRPV1. Similarly, mechanical hyperalgesia induced by dihydroxyphenylglycine (DHPG), an agonist for Group I metabotropic glutamate receptors (mGlu1/5), in the masseter was attenuated by AMG9810. DHPG-induced mechanical hyperalgesia was suppressed by pretreatment with a decoy peptide that disrupted interactions between TRPV1 and AKAP150. DHPG also upregulated p-S800 of TRPV1during which DHPG-induced mechanical hyperalgesia was prominent. Electrophysiological measurements in TG neurons demonstrated that TRPV1 sensitivity was enhanced by pretreatment with DHPG, and this was prevented by a PKC, but not by a PKA, inhibitor.

## Conclusions

Our data suggest that activation of both NMDA receptors and mGlu1/5 in masseter afferents invokes phosphorylation of TRPV1 serine residues including S800, and that phosphorylation-induced sensitization of TRPV1 is involved in masseter mechanical hyperalgesia. These data support a role of TRPV1 as an integrator of glutamate receptor signaling in trigeminal muscle nociceptors.

